# Sphingosine 1 Phosphate (S1P) Receptor 1 Is Decreased in Human Lung Microvascular Endothelial Cells of Smokers and Mediates S1P Effect on Autophagy

**DOI:** 10.3390/cells10051200

**Published:** 2021-05-14

**Authors:** Khushboo Goel, Erica L. Beatman, Nicholas Egersdorf, April Scruggs, Danting Cao, Evgeny V. Berdyshev, Kelly S. Schweitzer, Irina Petrache

**Affiliations:** 1 Department of Medicine, Division of Pulmonary Sciences and Critical Care Medicine, University of Colorado School of Medicine, Aurora, CO 80045, USA; khushboo.goel@cuanschutz.edu; 2 Department of Medicine, Division of Pulmonary, Critical Care, and Sleep Medicine, National Jewish Health, Denver, CO 80206, USA; ebeatman@gmail.com (E.L.B.); egersdorfn@njhealth.org (N.E.); aprilscruggs11@gmail.com (A.S.); dc3542@cumc.columbia.edu (D.C.); berdysheve@njhealth.org (E.V.B.); schweitzerk@njhealth.org (K.S.S.); 3 Department of Medicine, Division of Pulmonary, Allergy, Critical Care and Sleep Medicine, Indiana University School of Medicine, Indianapolis, IN 46202, USA

**Keywords:** sphingolipids, lung microvascular endothelial cells, chronic obstructive pulmonary disease, autophagy, apoptosis

## Abstract

Destruction of alveoli by apoptosis induced by cigarette smoke (CS) is a major driver of emphysema pathogenesis. However, when compared to cells isolated from non-smokers, primary human lung microvascular endothelial cells (HLMVECs) isolated from chronic smokers are more resilient when exposed to apoptosis-inducing ceramide. Whether this adaptation restores homeostasis is unknown. To better understand the phenotype of HLMVEC in smokers, we interrogated a major pro-survival pathway supported by sphingosine-1-phosphate (S1P) signaling via S1P receptor 1 (S1P1). Primary HLMVECs from lungs of non-smoker or smoker donors were isolated and studied in culture for up to five passages. S1P1 mRNA and protein abundance were significantly decreased in HLMVECs from smokers compared to non-smokers. S1P1 was also decreased in situ in lungs of mice chronically exposed to CS. Levels of S1P1 expression tended to correlate with those of autophagy markers, and increasing S1P (via S1P lyase knockdown with siRNA) stimulated baseline macroautophagy with lysosomal degradation. In turn, loss of S1P1 (siRNA) inhibited these effects of S1P on HLMVECs autophagy. These findings suggest that the anti-apoptotic phenotype of HLMVECs from smokers may be maladaptive, since it is associated with decreased S1P1 expression that may impair their autophagic response to S1P.

## 1. Introduction

Cigarette smoking (CS) is the leading cause of preventable disease and death in the United States (US), causing over 480,000 deaths annually [1]. Of the 36.5 million smokers in the US, 16 million suffer from smoking-related lung diseases, primarily chronic obstructive pulmonary disease (COPD), which is the 4th leading cause of death [2,3]. Despite decades of research, the complex effects of CS on lung health are not fully elucidated and, as a result, key therapeutic targets remain to be discovered.

One of the main phenotypes of COPD is emphysema, the hallmark of which is the destruction of gas exchange surfaces consisting of alveolar epithelial cells and lung microvascular endothelial cells (LMVECs) [2,4]. LMVECs are a crucial structural component of the alveolar capillary membrane and play an important role in gas exchange, host defense (e.g., by regulating the barrier function during inflammation), and injury repair (by promoting angiogenesis) [5,6]. Small molecules inhaled from CS are absorbed across the lung epithelium into circulation and can directly injure microvascular endothelial cells [7,8,9]. A key mechanism of CS-induced LMVEC injury and emphysema-like disease is the accumulation of ceramide, a bioactive sphingolipid that triggers endothelial cell apoptosis [10,11]. We have previously demonstrated that primary human LMVECs (HLMVECs) isolated from non-smoking donors obtained from a commercial source (Lonza, Walkersville, MD, USA) responded to long-chain palmitoyl ceramide (C16:0) by increasing endoplasmic reticulum stress and mitochondria depolarization, resulting in apoptosis [12]. In comparison, HLMVECs from donors with a history of chronic exposure to CS were more resistant to apoptosis, responding to palmitoyl ceramide with robust autophagy [12].

Autophagy is a common cellular response to stress, whereby unnecessary or dysfunctional cellular components are degraded and recycled in order to promote repair and survival [13,14,15,16]. Autophagy is a dynamic multi-step process (flux) that includes enveloping targeted constituents by a newly formed double membrane autophagosome that has to fuse with lysosomes for target delivery and degradation; these steps can be gauged by the abundance of select markers such as microtubule-associated protein 1A/1B light chain 3B (LC3B1/2), sequestosome-1 (SQSTM1/p62), and beclin1 [14,15]. It is now well established that COPD lungs exhibit increased autophagy, but its capability in resolving CS-induced lung injury is hampered by impairment in the completion of the autophagic flux or by the persistence of injury that overwhelms the cells’ repair capacity. Moreover, it is unclear if smokers’ HLMVEC adaptation to pro-apoptotic injury will restore homeostasis or will result in a dysfunctional phenotype that may contribute to further physiologic abnormalities in COPD, such as ventilation perfusion mismatch or chronic inflammation.

**Figure 1:**
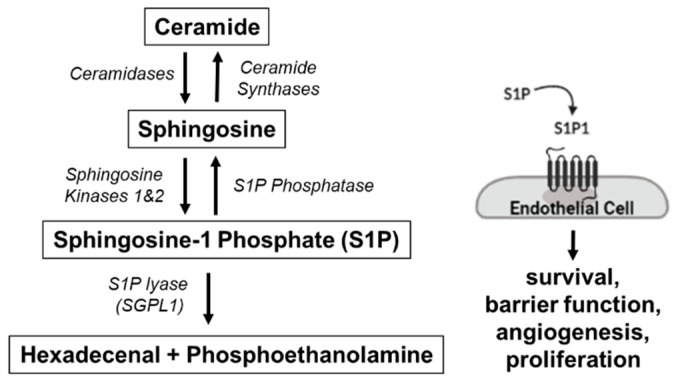
Sphingosine Metabolism. Sphingosine, produced by ceramide hydrolysis via ceramidase, is phosphorylated by sphingosine 1 kinase to generate sphingosine 1 phosphate (S1P), which promotes endothelial cell survival via S1P receptor 1 (S1P1). S1P is irreversibly degraded by S1P lyase (SGPL-1).

To better understand the smoker’s HLMVEC phenotype, we set out to interrogate its sphingosine-1-phosphate (S1P) signaling, a well-established robust endothelial pro-survival pathway [17]. S1P, a sphingolipid metabolite, exerts these effects by signaling either inside-out (not requiring receptors) or outside-in by engaging one of its five G protein-coupled S1P receptors (Figure 1). Of these, S1P receptor 1 (S1P1) is highly expressed on lung endothelium where it promotes survival and barrier function [18,19]. Given our previous findings of pro-survival adaptation of HLMVECs in smokers, we hypothesized that these cells will exhibit increased S1P–S1P1 signaling. Using primary HLMVECs isolated from donor lungs within 24 h of their surgical collection and maintained in tissue culture conditions for up to five passages, we found that S1P1 was in fact downregulated in cells of smokers compared to non-smokers. Using knockdown approaches, we also describe an important role for S1P1 in HLMVEC autophagic flux.

## 2. Materials and Methods

### 2.1. Cell Isolation and Culture

Human lungs were obtained through the Human Lung Tissue Consortium at National Jewish Health (NJH), Denver, CO that procures lungs from the Donor Alliance or the International Institute for the Advancement of Medicine. Lungs were donated but unused for transplantation from subjects known to be previously healthy that died of non-pulmonary causes and had no evidence of systemic infection or prior lung diseases. All protocols were approved by the Institutional Review Board at NJH. HLMVECs were isolated from de-identified donor lungs within 24 h of their surgical collection, based on the protocol outlined by Comhair et al. [20]. Briefly, after removal of lung pleura and the outermost layer of tissue, the tissue was digested and compressed to expel cells into a Petri dish with endothelial MCDB 107 growth medium (Sigma-Aldrich; M-6395; St. Louis, MO, USA), which is enriched with heparin (90 mg/L), endothelial growth supplement (100 mg/L), 10% fetal bovine serum (FBS), and 1% penicillin/streptomycin/fungizone. The suspension was filtered through a series of sterile nylon cell strainers (100 µm, 70 µm, and 40 µm pore diameters) in order to remove undigested tissues (100 µm, 70 µm) and to trap and collect cells (40 µm), which include microvascular endothelial cells [21]. The cells were cultured in MCDB 107 media in fibronectin-coated (CalBioChem; LS004176; San Diego, CA, USA) cell culture flasks at 37 °C, 5% CO_2_ with 90% humidity. To ensure that subsequent passages were highly enriched with endothelial cells, we performed an additional step of positive selection using anti-CD31 antibody-coated magnetic beads (Invitrogen; 11155D; Carlsbad, CA, USA) prior to plating cells into passage 2.

For indicated select experiments, primary HLMVECs were obtained from a commercial source (Lonza; CC2527) and were maintained in complete culture medium EGM-2 (Lonza; CC3156) supplemented with its specific Bullet-Kit (Lonza; CC4147). The smoking status, age, and gender of the donor were provided by the supplier. Cells from Lonza were used in experiments where SGPL-1 and S1P1 were silenced.

All experiments in HLMVECs were performed between passages 2 and 5.

### 2.2. Animal Studies

Animal studies were approved by the Institutional Animal Care and Use Committee (IACUC) of Indiana University and NJH. DBA/2J mice (3 months old; female; n = 4; Jackson Laboratory, Bar Harbor, ME, USA) or C57Bl/6 mice (3 months old; female; n = 3; Charles River, Wilmington, MA, USA) were exposed to ambient air control (AC) or to CS for 6 months. CS exposure consisted of 11% mainstream and 89% side stream and was delivered at a concentration of 100 mg/m^3^ total particulate matter, as previously described [22,23]. AC mice underwent a similar sleep cycle disruption and stimulation via handling. Lungs were harvested 24 h following the last CS exposure and processed as previously described [24].

### 2.3. Protein Isolation and Western Blotting

To extract proteins, cells were incubated with lysis buffer comprised of RIPA buffer (Sigma-Aldrich; R0278), PhosStop (Sigma-Aldrich; 4906837001), and Complete (Sigma-Aldrich; 4693116001) tablets, which contain protease inhibitor cocktails, followed by sonication and centrifugation for 10 min at 4 °C to collect the supernatant. Equal protein amounts, as determined by the Pierce bicinchoninic acid assay protein analysis (Thermo Fisher; 23225; Waltham, MA, USA), were separated by SDS-PAGE and transferred onto a polyvinylidene difluoride membrane (EMD Millipore; IPLF10100; Burlington, MA, USA), followed by routine Western immunoblotting. Blots were washed with TBS + 0.1% Tween-20 (TBST), blocked in TBST with 5% BSA solution, and incubated overnight with primary antibodies as follows: S1P1 (Novus Biological; NB120-11424; Centennial, CO, USA; 1:1000), beclin1 (Abcam; Ab207612; Cambridge, UK; 1:2000), LC3B (Sigma-Aldrich; L7543; 1:20,000–1:40,000), or p62 (Abnova; H00008878-M01; Taipei, Taiwan; 1:10,000). Blots were then washed and incubated with HRP-conjugated secondary antibodies to mouse (Sigma-Aldrich; NA931V; 1:10,000) or rabbit (Sigma-Aldrich; NA9340V; 1:10,000). Immune complexes were detected using enhanced chemiluminescence, quantified by densitometry (ImageJ software; version 1.53i; Bethesda, MD, USA) and normalized using specific loading controls vinculin (Abcam; Ab18058; 1:5000) or beta actin (Abcam; Ab207612; 1:2000).

### 2.4. RNA Isolation, Reverse Transcription, and Real-Time Quantitative Polymerase Chain Reaction (RTqPCR)

RNA isolation was performed using the RNEasy Plus Micro kit (Qiagen; 74034; Germantown, MD, USA) following the manufacturer’s instructions. Reverse transcription followed by RTqPCR was performed on the StepOnePlus System (Thermo Fisher; version 2.3) using Taqman gene expression assays. Human *18S* (H18S) was used as the reference/housekeeping gene (Thermo Fisher; 4333760F; Lot 1705040). The following primers were used: *S1P1* (Thermo Fisher; Hs00173499; Lot 1713063), *S1P2* (Thermo Fisher; APNKVD; Lot P190823-005 A04), *ACTA2* (Thermo Fisher; Hs004268358_g1; Lot 1839587), and *SGPL-1* (Thermo Fisher; Hs00187407_m1; Lot 1347476). Relative RNA expression was quantified as 2^−∆∆CT^ as previously described [25].

### 2.5. Immunohistochemistry

Sections of 3 µm thickness from paraffin-embedded lungs were heated in the oven at 60 °C, followed by deparaffinization in CitriSolv Hybrid Solvent (Thermo Fisher; 04-355-121) and serial washes of 100%, 95%, and 70% ethanol solutions. Antigen retrieval was performed in a pressure cooker with citric acid-based Antigen Unmasking Solution (Vector; H-3300; Burlingame, CA, USA; 1:100). The tissue sections were then incubated in 0.3% hydrogen peroxide (Sigma-Aldrich; H325-100; lot 201276) for 1 h, blocked in horse serum (Vector; PK-6200), incubated with S1P1 primary antibody (Novus Biological; NB120-11424; 1:50) overnight at 4 °C, followed by biotinylated anti-rabbit secondary antibody (Vector; BA-1000; 1:250) for 1 h at room temperature. The technical negative control was lung tissue stained exclusively with secondary antibodies. The sections were then incubated with avidin–biotin complex (Vector; PK-4000) followed by DAB substrate (Vector; SK-4100). Images were captured at 20X using a Nikon 80i microscope and analyzed blinded to the identity of the slides.

### 2.6. Immunofluorescence

Deparaffinization and antigen retrieval were performed as described for immunohistochemistry. The sections were blocked with 10% normal goat serum (Vector; S-1000-20) and 0.1% Triton-X solution (Sigma-Aldrich; X100-100 mL) in PBS and incubated with primary antibodies overnight at 4 °C: S1P1 (Novus Biological; NB120-11424; 1:70) and CD31 (R&D Systems; BBA7; Minneapolis, MN, USA; 1:250–1:500). Sections were then incubated with their corresponding secondary antibodies: goat anti-rabbit conjugated to Alexa Fluor 568 (Invitrogen; A11036; Lot 1704462; 1:200–1:250) and/or goat anti-mouse conjugated to Alexa Fluor 488 (Invitrogen; A21121; Lot 1889303; 1:200). The technical negative control was lung tissue stained exclusively with secondary antibodies. TrueView Autofluorescence Quenching Kit was applied per manufacturer’s instructions (Vector; SP-8500-15). The sections were mounted with Prolong Gold with DAPI mounting media (Thermo Fisher; P36931). Images were captured using a Nikon 80i microscope and analyzed blinded to the identity of the slides. Five random quadrants with primarily alveolar tissue per slide were imaged at 20 X. Image analysis was performed with ImageJ software using both manual and digital count to quantify S1P1 and CD31 co-localization.

### 2.7. siRNA Transfection

For transfection experiments, we used HLMVECs purchased from Lonza that were obtained from non-smoker donors. Cells were incubated in AC extracts for 72 h in EGM-2 media prior to assessment. This extract was prepared by bubbling room air for 2 min, each into 20 mL of PBS (100% *v*/*v*). AC extracts were then adjusted to pH 7.4 and filtered with 0.22 µm filters, as previously described [22]. Cells were transfected at 80% to 90% confluency with small interfering RNA (siRNA) siS1P1 (Dharmacon; M-003655-02-005; Lafayette, CO, USA; 20 µM) and/or siSGPL-1 (Dharmacon; E-008747-00-0005; 20 µM) for 72 h, using lipofectamine RNAiMAX (Invitrogen; 13778150) following the manufacturer’s specifications. Control cells were transfected with non-targeting (NT) antisense inhibitors (Dharmacon; D-001206-13-20; 20 µM; 24 h). Transfection efficiencies were verified with RTqPCR.

### 2.8. Quantitative Sphingolipid Determination

Lipid extraction, P_i_ labeling with NH_4_-molybdate, and total lipid phosphorus (P_i_) measurements were performed as previously described and were utilized to calculate sphingolipid concentrations [10]. Sphingolipid analyses were performed via combined liquid chromatography–tandem mass spectrometry (LC–MS/MS), using the API4000 Q-trap hybrid triple quadrupole linear ion-trap mass spectrometer (Applied Biosystems/Sciex; Framingham, MA, USA) equipped with turbo ion spray ionization source and Agilent 1100 series liquid chromatography as a front end (Agilent Technologies; Wilmington, DE, USA) as previously described [26].

### 2.9. Statistical Analysis

Statistical analysis was performed using GraphPad Prism software (version 7.0; La Jolla, CA, USA). Differences between groups were compared using 2-tailed unpaired Student’s *t*-test, 1-way ANOVA with Tukey’s multiple comparisons test, or Pearson correlation analysis. The data were expressed as mean ± SEM. Statistical difference was accepted at a *p* value < 0.05.

## 3. Results

### 3.1. Effect of Smoking on S1P1 Expression and Abundance in HLMVECs

**Figure 2:**
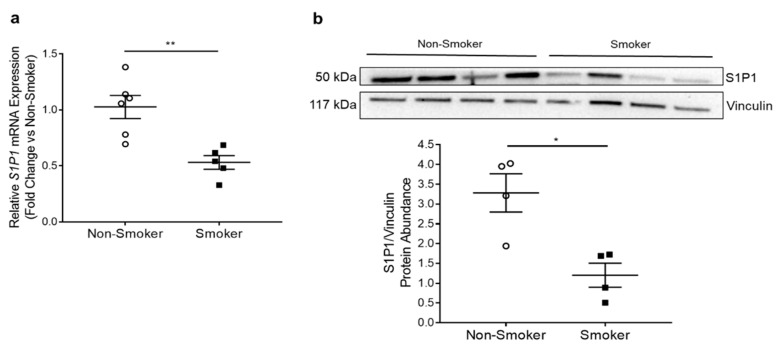
S1P1 in HLMVECs from Non-Smokers and Smokers. (**a**) Relative *S1P1* mRNA expression in HLMVECs isolated from non-smoker (n = 6) and smoker (n = 5) donors measured by RTqPCR, normalized to the housekeeping gene human *18S* (H18S) using 2^−∆∆CT^ method and expressed as a fold-change versus control (non-smoker). ** *p* = 0.0035; 2-tailed unpaired Student’s *t*-test. (**b**) Representative Western blot and densitometry analysis of S1P1 protein abundance relative to vinculin loading control in non-smoker and smoker HLMVECs (n = 4; 20 µg protein/well). * *p* = 0.01; 2-tailed unpaired Student’s *t*-test. All graphs show data points, each representing an individual donor; horizontal lines are mean ± SEM.

We isolated primary HLMVECs from lungs of donors using a method adapted from Comhair et al. [20]. We defined donors as non-smokers if they had never smoked or had a remote and minimal smoking history or as smokers if they were active smokers until their death. Demographic and brief clinical characteristics of deidentified donors whose lungs were used for indicated experiments are listed in Table S1. We quantified *S1P1* mRNA expression by RTqPCR in HLMVECs isolated from six non-smokers and five smokers. We found that smokers’ HLMVECs had significantly decreased *S1P1* mRNA expression (*p* = 0.0035) (Figure 2a). We next quantified S1P1 protein abundance by Western blotting on HLMVEC protein lysates, which also exhibited a significant decrease in S1P1 in smokers (*p* = 0.01) (Figure 2b). To investigate if other S1P receptors were affected, we focused on S1P receptor 2 (S1P2), whose downstream signaling may counteract the pro-survival signaling initiated by S1P1, inducing endothelial cell dysfunction and transdifferentiation, such as the upregulation of smooth muscle gene alpha-actin [27]. There was no significant difference in *S1P2* mRNA expression between non-smokers and smokers (Figure S1a) or in *ACTA-2* mRNA expression, which encodes alpha-2 smooth muscle actin (Figure S1b).

**Figure 3:**
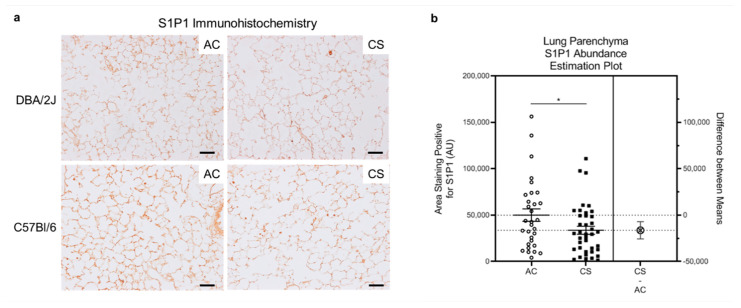
S1P1 in Lung Parenchyma of Mice Chronically Exposed to Cigarette Smoke (CS). (**a**) Representative images of S1P1 abundance (brown) in the alveolar regions of mouse lungs determined by immunohistochemistry (IHC) in DBA/2J mice (n = 4) or C57Bl/6 mice (n = 3) exposed to ambient air control (AC) or CS for 6 months. Note the overall decreased S1P1 staining in CS mice. IHC was performed on paraffin-embedded lung tissue sections with anti-S1P1 antibody (1:50). Scale bar is 50 µm. (**b**) Quantitative assessment of the alveolar area staining positive for S1P1 by IHC in DBA/2J mice (n = 4) exposed to AC or CS for 6 months, obtained by automated analyses of images captured by an operator blinded to the identity of the slides. Each data point represents average from a lung parenchyma image; horizontal lines are mean ± SEM. * *p* = 0.04; 2-tailed unpaired Student’s *t*-test with Welch correction.

Since the study of HLMVECs was performed ex vivo on cultured cells following lung homogenization and single cell isolation, the results may not fully recapitulate in vivo phenotypes. We therefore investigated S1P1 abundance in lung parenchyma in situ following chronic smoking in mouse and human tissues. In a model of chronic exposure, DBA/2J (n = 4) or C57Bl/6 (n = 3) mice were exposed to AC or CS for 6 months. We performed immunohistochemistry for S1P1 in the mouse lung, focusing on the distal lung where LMVECs are primarily found. Consistent with the results obtained in cultured HLMVECs, alveolar parenchyma from CS-exposed mice had decreased S1P1 abundance (Figure 3a,b). We next performed immunofluorescence for S1P1, co-staining for the endothelial cell marker CD31, on human lung alveolar tissue from 11 non-smokers and 11 smokers (donor details provided in Table S1) obtained from the NJH Human Lung Tissue Consortium. We analyzed one slide per patient and five quadrants per slide, focusing primarily on the alveolar areas. We calculated the proportion of endothelial cells that were positive for both CD31 staining (green) and S1P1 staining (red). As S1P1 can be either located on the surface of the endothelial cell plasma membrane or internalized intra- cellular, we counted cells that displayed both green (CD31) and red (S1P1) staining in close proximity to each other and to a nucleus (DAPI-positive, stained in blue) (Figure S2a) [28,29]. There was high variability in the abundance of S1P1-expressing endothelial cells in these tissues, without appreciable differences between non-smokers and smokers (Figure S2b).

### 3.2. S1P1 Role in HLMVEC Autophagy

**Figure 4:**
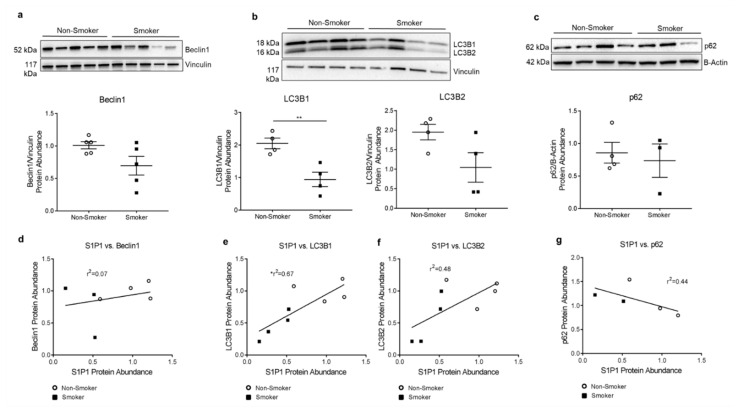
Autophagy in HLMVECs from Smokers. (**a**–**c**) Representative Western blot and densitometry analysis with vinculin (**a**,**b**) or actin (**c**) as loading control in non-smoker and smoker HLMVECs of (**a**) beclin1 abundance, 25 µg (*p* = 0.07); (**b**) LC3B1 and LC3B2, 20 µg protein per well (** *p* = 0.007. LC3B2 *p* = 0.078.); (**c**) p62, 20 µg protein per well (*p* = 0.69). All graphs (**a**–**c**) show data points, each representing an individual donor; horizontal lines are mean ± SEM. Two-tailed unpaired Student’s *t*-test used for statistical analysis. (**d**–**g**) Correlations between (**d**) S1P1 and beclin1. r^2^ = 0.07; *p* = 0.55. (**e**) S1P1 and LC3B1. r^2^ = 0.67; * *p* = 0.01. (**f**) S1P1 and LC3B2. r^2^ = 0.48; *p* = 0.06. (**g**) S1P1 and p62. r^2^ = 0.44; *p* = 0.22. All graphs (**d**–**g**) show data points, each representing an individual donor. Only HLMVECs which were matched by donor and passage number were included in correlation analysis. Each densitometry value was first normalized to the average control (non-smoker) densitometry obtained from its respective Western blot. Pearson’s correlation coefficients with two-tailed *p*-values used for statistical analyses.

We have previously shown brisk autophagic response to apoptotic stress (i.e., ceramide augmentation) in HLMVECs from smokers [12]. However, whether the baseline autophagy in these cells may be altered by chronic smoking remains unclear. We performed immunoblotting on protein lysates obtained from non-smoker and smoker HLMVECs for beclin1, LC3B, and p62. We noted decreases or trends towards decreases in the abundance of autophagy markers beclin1 (*p* = 0.07), LC3B1 (*p* = 0.007), and LC3B2 (*p* = 0.078) in smokers’ HLMVECs compared to non-smokers (Figure 4a,b). These changes were not accompanied by significant differences in markers of autophagic flux, such as the ratio of LC3B2/LC3B1 or the abundance p62 in smoker compared to non-smoker cells (Figure 4b,c). Given the high variability among cells from individual human donors, we analyzed data matched by individual HLMVEC donor and passage number. We again noted significant direct correlations between S1P1 and LC3B1 (r^2^ = 0.67; *p* = 0.01) a trend for correlation with LC3B2 (r^2^ = 0.48; *p* = 0.06), and a trend for inverse correlation with p62 protein abundance (r^2^ = 0.44; *p* = 0.22) (Figure 4d–g). As such, we next investigated the mechanistic role of S1P1 in HLMVEC baseline autophagy rates (in the absence of stress).

**Figure 5:**
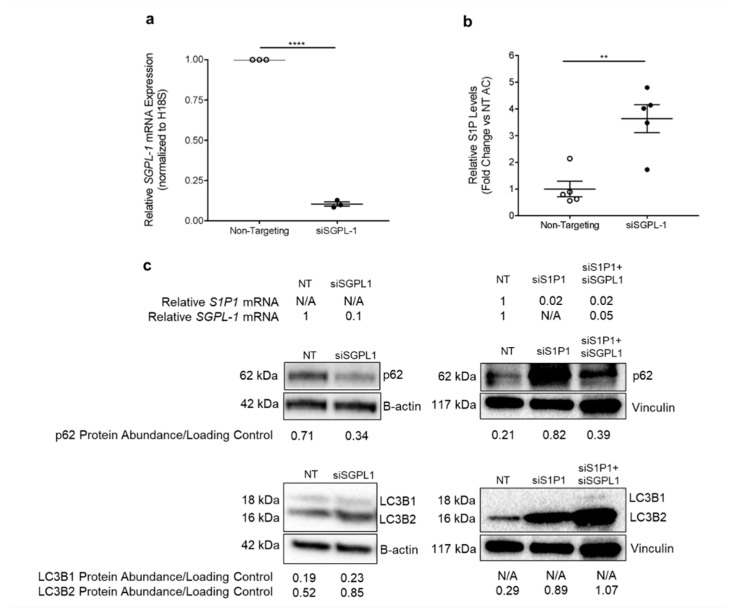
S1P–S1P1 Signaling Effect on HLMVEC Autophagy. (**a**) Relative SGPL-1 mRNA expression in non-smoker HLMVECs (Lonza; n = 3) measured by RTqPCR, normalized to the housekeeping gene human 18S (H18S) using the 2^−∆∆CT^ method, expressed as fold-change versus mean control (non-targeting siRNA) expression. **** *p* < 0.0001; 2-tailed unpaired Student’s *t*-test. (**b**) Relative S1P levels in non-smoker HLMVECs (Lonza; n = 5) transfected with siSGPL-1 for 72 h, measured by mass spectrometry. ** *p* = 0.002; 2-tailed unpaired Student’s *t*-test. All graphs show data points, each representing an individual donor; horizontal lines are mean ± SEM. (**c**) Representative Western blots of LC3B and p62 protein abundance in non-smoker HLMVECs (Lonza; n = 1) transfected with non-targeting (NT) siRNA, siSGPL-1 (10 µg protein/well), siS1P1 (20 µg protein/well), or co-transfection with siS1P1 and siSGPL-1 (20 µg protein/well) for 72 h. B-actin and vinculin were used as loading controls. Written above is the relative expression of *S1P1* and *SGPL-1* mRNA after NT or siRNA transfection, by RTqPCR and normalized to housekeeping gene *H18S* using the 2^−∆∆CT^ method. Below is the associated densitometry analysis, calculated as the ratio of protein abundance to loading control.

Since S1P supports HLMVEC survival, we hypothesized that increasing S1P signaling in HLMVECs promotes the completion of autophagic flux via S1P1. Exogenous S1P has a short half-life [30] and S1P agonists such as FTY720 have multiple non-specific effects [31]. However, when generated intracellularly, S1P can be transported to exert outside-in signaling via its receptors. Thus, in order to sustainably increase S1P levels, we inhibited its degradation by the S1P lyase (an enzyme encoded by *SGPL-1*) (Figure 1). This method also allowed us to test the role of both outside-in and intracellular signaling of S1P. We transfected non-smoker HLMVECs with SGPL-1 siRNA (siSGPL-1), using non-targeting (NT) siRNA as the control. siSGPL-1 transfection expectedly decreased *SGPL-1* mRNA expression by 90% (Figure 5a) and increased intracellular S1P levels by more than two-fold compared to the NT control, as measured by mass spectrometry (*p* = 0.002) (Figure 5b). HLMVECs transfected with siSGPL-1 had increased conversion of LC3B1 to LC3B2 and decreased p62 abundance compared to the NT control, consistent with the activation and completion of the autophagic flux (Figure 5c).

We next investigated whether S1P’s effects on autophagic flux were dependent on S1P1, using a knockdown approach. We transfected non-smoker HLMVECs with either S1P1 siRNA (siS1P1) alone or both siSGPL-1 and siS1P1 for 72 h, which decreased *SGPL-1* and *S1P1* mRNA expression by 95% and 98%, respectively, when compared to the NT control (Figure 5c). Interestingly, transfection with siS1P1 alone and co-transfection with siS1P1 and siSGPL-1 increased the conversion of LC3B1 to LC3B2, as measured by Western blotting. HLMVECs co-transfected with siSGPL-1 and siS1P1 showed increased p62 compared to NT control cells; notably, transfection with siS1P1 alone also increased the accumulation of p62 (Figure 5c). Taken together, these results suggest that S1P1 is necessary for S1P signaling to augment and complete autophagic flux.

## 4. Discussion

We identified significant baseline differences of decreased abundance of S1P1 in HLMVECs isolated from chronic smokers that are consistent with changes in the lung parenchyma of mice chronically exposed to cigarette smoke. We also identified that S1P1 is necessary for S1P signaling of autophagy in HLMVECs.

To our knowledge, this is the first study to show that HLMVECs isolated from chronic smokers have decreased S1P1 mRNA expression and protein abundance compared to non-smokers. The levels of S1P1 abundance in situ in areas of lung parenchyma that are enriched in alveoli and therefore in LMVECs were similarly decreased by chronic CS exposure in two mouse strains (DBA/2J and C57Bl/6). However, in human lung parenchyma from both non-smoker and smoker donors, there was a highly variable expression of S1P1 abundance in cells co-expressing the endothelial cell marker CD31. Although individual variability is expected in a small sample size, HLMVECs isolated from the same lungs did show consistent decreases in S1P1 in smokers. Other potential explanations include: differences in the antibodies used, the clinical presentation of the donors (e.g., trauma, cardiac arrest), as well as effects of interventions surrounding organ procurement (e.g., duration and level of ventilation prior to explantation and duration of organ harvesting and transportation) that may have temporarily masked the chronic effects of smoking on HLMVECs. These limitations illustrate the challenges of research that rely on a small sample size of difficult to obtain human specimens due to the paucity of donated lungs. 

Since the differences in S1P1 expression were consistent for several passages in culture, we suspect that the lower S1P1 expression may have epigenetic origin, although we cannot exclude transcriptional or post-translational mechanisms at play. Our results suggest that the decreased S1P1 protein abundance is associated with a decrease in S1P1 transcription. The activation of S1P1 by its ligand S1P at the plasma membrane leads to rapid receptor internalization via clathrin-mediated endocytosis and is then either recycled back to the plasma membrane or undergoes ubiquitination and proteasome-mediated degradation [28,29]. The phosphorylation of S1P1 at its serine and tyrosine residues has been shown to be a key mechanism of promoting receptor internalization and subsequent degradation, thus regulating S1P1′s endothelial cell-surface abundance [32,33,34]. Several mechanisms have been implicated in the transcription of S1P1, depending on the cell-type. For example, Igarashi et al. showed that vascular endothelial growth factor (VEGF) induces the expression of *S1P1* in bovine aortic endothelial cells via protein tyrosine kinase and PKC signaling pathways [35]. Josipovic et al. showed that the LISPR1 promotes *S1P1* expression in human umbilical endothelial cells, possibly by facilitating RNA polymerase II binding [36]. Carlson et al. showed that Kruppel-like factor 2 deficiency was associated with impaired *S1P1* expression in thymocytes [37]. Future studies about the precise origin of decreased S1P1 following chronic smoking are important to pursue, since S1P–S1P1 signaling plays an important role in HLMVEC survival [11].

Furthermore, although CS is well-described as being injurious to lung endothelial cells via the accumulation of pro-apoptotic ceramide, the alterations induced by chronic CS exposure in the surviving cells’ phenotype may affect their cell function [12]. We have previously shown that HLMVECs from smokers, despite having reduced S1P at baseline, respond to pro-apoptotic stress with enhanced autophagy signaling [12]. Despite decreased baseline S1P1 in HLMVECs from smokers, the levels of autophagy effectors beclin1, LC3B, and p62 did not indicate the activation of autophagy during homeostatic conditions. However, we noted trends towards an association between S1P1 abundance and that of beclin1, LC3B1/2, and p62, which reached statistical significance for LC3B1. To our knowledge, correlations between S1P1 and autophagy markers have not been previously reported. This finding may have implications in the ability of HLMVECs chronically exposed to injurious insults such as CS to undergo quality-control and/or stress-induced autophagy. Indeed, in our previous report, we showed that baseline Akt hyper-phosphorylation in HLMVECs from chronic smokers contributed to a phenotype that was characterized by resistance to apoptosis with brisk autophagy induction by exogenous ceramide [12]. Of note, this previous work was performed in HLMVECs obtained from a commercial source, whereas the current study was performed in HLMVECs isolated from donor lungs based on the protocol by Comhair et al. that enhances the purity of lung endothelial cells, as evidenced by morphology, gene expression profile, and functional assessment [20]. The cell culture media also differed, in that for the former study we used proprietary basal media with a bullet kit (CC3156, CC4147) with human fibroblast growth factor and 5% FBS as recommended by the vendor, whereas for the current study we used MCDB-105 media with endothelial cell growth supplement and 10% FBS, following published protocols. 

We determined that S1P1 was required for S1P to promote the completion of autophagic flux during basal growth conditions, since silencing S1P1 had a major effect on attenuating the effect of increased S1P, as shown by the accumulation of p62. This finding advances our mechanistic understanding of how the augmentation of S1P promotes endothelial cell survival and autophagy [11,38,39,40]. Our results are consistent with our previous work in HLMVECs, where we have shown that the overexpression of glucosylceramide synthase, which led to increased S1P levels, was associated with improved autophagic flux [40].

An alternative hypothesis is that because of the loss of S1P1, S1P might bind to other S1P receptors to modulate autophagy. We did not evaluate HLMVECs’ expression of all S1P receptors (apart from S1P2) in this report and acknowledge that further studies performing serial knockdown experiments would be required to elucidate their potential involvement in autophagy. In addition, smoker lungs may have abnormal expression of various S1P receptors in other cell types, with pathogenic implications. For example, although S1P–S1P2 signaling may counteract pro-survival S1P–S1P1 signaling in HLMVECs, S1P2 signaling may be beneficial in other cell-types; Chen et al. showed that in response to Pseudomonas aeruginosa-induced lung injury, S1P acted via S1P2 to promote alveolar repair by increasing the differentiation of alveolar type 1 to type 2 cells [27,41]. Nevertheless, our finding that silencing S1P1 alone in HLMVECs led to the accumulation of p62 highlights the key role of S1P1 in the completion of autophagic flux. These mechanistic experiments help interpret our previously reported findings of low S1P levels in HLMVECs from smokers [12], indicating that coupled with a low S1P1 expression, these cells may be hampered in their ability to complete autophagic flux during homeostatic conditions, when this process is required for the removal of damaged molecules and organelles, thus ensuring the “quality control” of the cell.

We believe our results may have therapeutic implications. We have previously identified that the balance between ceramide and S1P levels is crucial to determining HLMVEC outcome, and that excessive inhibition of ceramide may have an untoward outcome of depleting its metabolite sphingosine and consequently decreasing levels of the pro-survival S1P [10,11]. Therefore, S1P-based therapy to promote HLMVEC survival is tempting, but its short half-life of ~15 min in vivo, whether due to dephosphorylation or intracellular degradation, renders treatment with S1P impractical [30]. As such, researchers have turned to reagents which activate S1P1 directly. We previously demonstrated that administering the S1P1 agonist SEW2871 counteracted VEGFR inhibitor-induced lung apoptosis [11]. Camp et al. showed that the S-enantiomer, 1-phosphonate, and 2-enephosphonate analogs of FTY720, which are more selective S1P1 agonists, enhance lung endothelial cell survival [31]. We have also shown that introducing FTY phosphonate analogs in HLMVECs attenuated the deleterious effects of CS extract on barrier function [42]. Our study provides additional mechanistic context in support of S1P1-focused therapies to enhance the quality of HLMVEC autophagy. It remains to be demonstrated if the decreased S1P1 expression in chronic smoking could hamper the efficiency of these drugs.

## 5. Conclusions

Chronic CS exposure is associated with decreased S1P1 in HLMVECs, which may affect their potential to appropriately undergo autophagic flux in response to S1P. In turn, these phenotypic changes could impair the function of HLMVECs as key contributors to gas exchange and lung repair, which could lead to lung remodeling in chronic smokers, such as emphysema or pulmonary hypertension.

## Data Availability

The data presented in this study are available in the article and supplementary material.

## Supplementary Materials

The following are available online at https://www.mdpi.com/article/10.3390/cells10051200/s1, Table S1: Characteristics of Donor HLMVECs, Figure S1: *S1P2* and *ACTA-2* mRNA Expression in HLMVECs.

## References

[1] National Center for Chronic Disease Prevention and Health Promotion (US) Office on Smoking and Health (2014). The Health Consequences of Smoking-50 Years of Progress: A Report of the Surgeon General.

[2] Tuder R.M., Petrache I. (2012). Pathogenesis of chronic obstructive pulmonary disease. J. Clin. Investig..

[3] Cornelius M.E., Wang T.W., Jamal A., Loretan C.G., Neff L.J. (2020). Tobacco Product Use Among Adults—United States, 2019. Morb. Mortal. Wkly. Rep..

[4] Suzuki Y., Yoshimura K., Uto T., Sato J., Imokawa S., Suda T. (2016). Morphological changes in small pulmonary vessels are associated with severe acute exacerbation in chronic obstructive pulmonary disease. Int. J. Chronic Obstr. Pulm. Dis..

[5] King J., Hamil T., Creighton J., Wu S., Bhat P., McDonald F., Stevens T. (2004). Structural and functional characteristics of lung macro- and microvascular endothelial cell phenotypes. Microvasc. Res..

[6] Garcia J.G. (2009). Concepts in microvascular endothelial barrier regulation in health and disease. Microvasc. Res..

[7] Boucher R.C., Johnson J., Inoue S., Hulbert W., Hogg J.C. (1980). The effect of cigarette smoke on the permeability of guinea pig airways. Lab. Investig..

[8] Low B., Liang M., Fu J. (2007). p38 mitogen-activated protein kinase mediates sidestream cigarette smoke-induced endothelial permeability. J. Pharmacol. Sci..

[9] Serikov V.B., Leutenegger C., Krutilina R., Kropotov A., Pleskach N., Suh J.H., Tomilin N.V. (2006). Cigarette smoke extract inhibits expression of peroxiredoxin V and increases airway epithelial permeability. Inhal. Toxicol..

[10] Petrache I., Natarajan V., Zhen L., Medler T.R., Richter A., Cho C., Hubbard W.C., Berdyshev E.V., Tuder R.M. (2005). Ceramide Upregulation Causes Pulmonary Cell Apoptosis and Emphysema. Nat. Med..

[11] Diab K.J., Adamowicz J.J., Kamocki K., Rush N.I., Garrison J., Gu Y., Schweitzer K.S., Skobeleva A., Rajashekhar G., Hubbard W.C. (2010). Stimulation of sphingosine 1-phosphate signaling as an alveolar cell survival strategy in emphysema. Am. J. Respir. Crit. Care Med..

[12] Petrusca D.N., Van Demark M., Gu Y., Justice M.J., Rogozea A., Hubbard W.C., Petrache I. (2014). Smoking exposure induces human lung endothelial cell adaptation to apoptotic stress. Am. J. Respir. Cell Mol. Biol..

[13] Galluzzi L., Vitale I., Aaronson S.A., Abrams J.M., Adam D., Agostinis P., Alnemri E.S., Altucci L., Amelio I., Andrews D.W. (2018). Molecular mechanisms of cell death: Recommendations of the Nomenclature Committee on Cell Death 2018. Cell Death Differ..

[14] Zhang X.-J., Chen S., Huang K.-X., Le W.-D. (2013). Why should autophagic flux be assessed?. Acta Pharmacol. Sin..

[15] Singh R., Letai A., Sarosiek K. (2019). Regulation of apoptosis in health and disease: The balancing act of BCL-2 family proteins. Nat. Rev. Mol. Cell Biol..

[16] Petrache I., Birukova A., Ramirez S.I., Garcia J.G.N., Verin A.D. (2003). The role of the microtubules in tumor necrosis factor-alpha-induced endothelial cell permeability. Am. J. Respir. Cell Mol. Biol..

[17] Spiegel S., Milstien S. (2003). Sphingosine-1-phosphate: An enigmatic signalling lipid. Nat. Rev. Mol. Cell Biol..

[18] Cartier A., Hla T. (2019). Sphingosine 1-phosphate: Lipid signaling in pathology and therapy. Science.

[19] Takuwa Y., Takuwa N., Sugimoto N. (2002). The Edg family G protein-coupled receptors for lysophospholipids: Their signaling properties and biological activities. J. Biochem..

[20] Comhair S.A., Xu W., Mavrakis L., Aldred M.A., Asosingh K., Erzurum S.C. (2012). Human Primary Lung Endothelial Cells in Culture. Am. J. Respir. Cell Mol. Biol..

[21] Tuder R.M., Marecki J.C., Richter A., Fijalkowska I., Flores S. (2007). Pathology of Pulmonary Hypertension. Clin. Chest Med..

[22] Schweitzer K.S., Johnstone B.H., Garrison J., Rush N.I., Cooper S., Traktuev D.O., Feng D., Adamowicz J.J., Van Demark M., Fisher A.J. (2011). Adipose stem cell treatment in mice attenuates lung and systemic injury induced by cigarette smoking. Am. J. Respir. Crit. Care Med..

[23] Cruickshank-Quinn C.I., Mahaffey S., Justice M.J., Hughes G., Armstrong M., Bowler R.P., Reisdorph R., Petrache I., Reisdorph N. (2014). Transient and persistent metabolomic changes in plasma following chronic cigarette smoke exposure in a mouse model. PLoS ONE.

[24] Clauss M., Voswinckel R., Rajashekhar G., Sigua N.L., Fehrenbach H., Rush N.I., Schweitzer K.S., Yildirim A.Ö., Kamocki K., Fisher A.J. (2011). Lung endothelial monocyte-activating protein 2 is a mediator of cigarette smoke-induced emphysema in mice. J. Clin. Investig..

[25] Livak K.J., Schmittgen T.D. (2001). Analysis of relative gene expression data using real-time quantitative PCR and the 2(-Delta Delta C(T)) Method. Methods.

[26] Justice M.J., Bronova I., Schweitzer K.S., Poirier C., Blum J.S., Berdyshev E.V., Petrache I. (2018). Inhibition of acid sphingomyelinase disrupts LYNUS signaling and triggers autophagy. J. Lipid Res..

[27] Skoura A., Hla T. (2008). Regulation of vascular physiology and pathology by the S1P2 receptor subtype. Cardiovasc. Res..

[28] Reeves P.M., Kang Y.-L., Kirchhausen T. (2016). Endocytosis of Ligand-Activated Sphingosine 1-Phosphate Receptor 1 Mediated by the Clathrin-Pathway. Traffic.

[29] Martínez-Morales J.C., Romero-Ávila M.T., Reyes-Cruz G., García-Sáinz J.A. (2018). S1P1 receptor phosphorylation, internalization, and interaction with Rab proteins: Effects of sphingosine 1-phosphate, FTY720-P, phorbol esters, and paroxetine. Biosci. Rep..

[30] Venkataraman K., Lee Y.-M., Michaud J., Thangada S., Ai Y., Bonkovsky H.L., Parikh N.S., Habrukowich C., Hla T. (2008). Vascular endothelium as a contributor of plasma sphingosine 1-phosphate. Circ. Res..

[31] Camp S.M., Chiang E.T., Sun C., Usatyuk P.V., Bittman R., Natarajan V., Garcia J.G.N., Dudek S.M. (2016). Pulmonary Endothelial Cell Barrier Enhancement by Novel FTY720 Analogs: Methoxy-FTY720, Fluoro-FTY720, and beta-Glucuronide-FTY720. Chem. Phys. Lipids.

[32] Chavez A., Schmidt T.T., Yazbeck P., Rajput C., Desai B., Sukriti S., Giantsos-Adams K., Knezevic N., Malik A.B., Mehta D. (2015). S1PR1 Tyr143 phosphorylation downregulates endothelial cell surface S1PR1 expression and responsiveness. J. Cell Sci..

[33] Oo M.L., Thangada S., Wu M.-T., Liu C.H., Macdonald T.L., Lynch K.R., Lin C.-Y., Hla T. (2007). Immunosuppressive and anti-angiogenic sphingosine 1-phosphate receptor-1 agonists induce ubiquitinylation and proteasomal degradation of the receptor. J. Biol. Chem..

[34] Watterson K.R., Johnston E., Chalmers C., Pronin A., Cook S.J., Benovic J.L., Palmer T.M. (2002). Dual regulation of EDG1/S1P(1) receptor phosphorylation and internalization by protein kinase C and G-protein-coupled receptor kinase 2. J. Biol. Chem..

[35] Igarashi J., Erwin P.A., Dantas A.P.V., Chen H., Michel T. (2003). VEGF induces S1P1 receptors in endothelial cells: Implications for cross-talk between sphingolipid and growth factor receptors. Proc. Natl. Acad. Sci. USA.

[36] Josipovic I., Pflüger B., Fork C., Vasconez A.E., Oo J.A., Hitzel J., Seredinski S., Gamen E., Zu Heringdorf D.M., Chen W. (2018). Long noncoding RNA LISPR1 is required for S1P signaling and endothelial cell function. J. Mol. Cell. Cardiol..

[37] Carlson C.M., Endrizzi B.T., Wu J., Ding X., Weinreich M.A., Walsh E.R., Wani M.A., Lingrel J.B., Hogquist K.A., Jameson S.C. (2006). Kruppel-like factor 2 regulates thymocyte and T-cell migration. Nature.

[38] Cuvillier O., Pirianov G., Kleuser B., Vanek P.G., Coso O.A., Gutkind J.S., Spiegel S. (1996). Suppression of ceramide-mediated programmed cell death by sphingosine-1-phosphate. Nature.

[39] Payne S.G., Milstien S., Spiegel S. (2002). Sphingosine-1-phosphate: Dual messenger functions. FEBS Lett..

[40] Koike K., Berdyshev E.V., Mikosz A.M., Bronova I.A., Bronoff A.S., Jung J.P., Beatman E.L., Ni K., Cao D., Scruggs A.K. (2019). Role of Glucosylceramide in Lung Endothelial Cell Fate and Emphysema. Am. J. Respir. Crit. Care Med..

[41] Chen Q., Rehman J., Chan M., Fu P., Dudek S.M., Natarajan V., Malik A.B., Liu Y. (2020). Angiocrine Sphingosine-1-Phosphate Activation of S1PR2-YAP Signaling Axis in Alveolar Type II Cells Is Essential for Lung Repair. Cell Rep..

[42] Schweitzer K.S., Chen S.X., Law S., Van Demark M., Poirier C., Justice M.J., Hubbard W.C., Kim E.S., Lai X., Wang M. (2015). Endothelial disruptive proinflammatory effects of nicotine and e-cigarette vapor exposures. Am. J. Physiol. Cell. Mol. Physiol..

