# Supplementary material for: Sphingosine 1 Phosphate (S1P) Receptor 1 Is Decreased in Human Lung Microvascular Endothelial Cells of Smokers and Mediates S1P Effect on Autophagy
Source: Cells. 2021 May 14;10(5):1200. doi: 10.3390/cells10051200 (PMC8156252; doi:10.3390/cells10051200)

Table S1. Characteristics of Donors**.** Clinical information for the human lung tissue samples obtained through the National Jewish Health Human Lung Tissue Consortium are listed. Non-smokers were defined as individuals without any smoking history or a very remote and minimal smoking history. Smokers were defined as individuals with active smoking history until their death. Primary lung microvascular endothelial cells were isolated from certain individuals and used for cell culture experiments, whereas the immunofluorescence (IF) experiments were performed on paraffin-embedded lung tissue sections. *Smoked 2-3 cigarettes/month for 3 years. **Smoked 1 cigarette/day for 1 year. ***Smoked marijuana, cocaine >20 years ago. x: Information is not applicable to the sample.

	Clinical Information			Corresponding Experiments		
				Cell Culture		Lung Tissue IF
No.	Donor Age	Donor Sex	Smoking History	Passage No.	Figure	Figure
**Non-Smoker**						
1	76	M	Non-smoker	2	2a; S1a,b.	x
2	15	M	Non-smoker	4	2a,b; 4a-g; S1a,b.	x
3	35	M	Non-smoker	3	2a,b; 4a-g; S1a,b.	x
4	77	F	Non-smoker	5	2a,b; 4a-g; S1a,b.	x
5	40	M	Remote*	4	2a; 4a,c, d, g; S1a,b.	x
6	59	F	Non-smoker	2	2a,b; 4a,b,d,e,f; S1a,b.	S2b
7	31	M	Remote**	x	x	S2b
8	79	F	Non-smoker	x	x	S2b
9	62	F	Non-smoker	x	x	S2b
10	61	F	Non-smoker	x	x	S2b
11	66	F	Non-smoker	x	x	S2b
12	56	M	Non-smoker	x	x	S2b
13	39	M	Non-smoker	x	x	S2b
14	Unknown	M	Non-smoker	x	x	S2b
15	76	F	Non-smoker	x	x	S2b
16	61	F	Non-smoker	x	x	S2b
**Smoker**						
1	55	M	>40 pack-years	5	2a,b; 4b,e,f; S1a,b.	x
2	55	F	30 pack-years	4	2a,b; 4a-g; S1a,b.	x
3	62	F	40 pack-years	3	2a,b; 4a-g; S1a,b.	x
4	52	M	Not specified	4	4a,c.	S2b
5	52	M	Smoked <1 ppd for decades***	3	4a.	S2b
				5	2a; S1a,b.	
6	59	F	1.5 pack-years	3	2a,b; 4a,b,d,e,f; S1a,b.	S2b
7	32	M	12.75 pack-years	x	x	S2b
8	61	F	0.5-1ppd for 30 years	x	x	S2b
9	55	F	30 pack-years	x	x	S2b
10	36	F	1-2ppd for 5-10 years	x	x	S2b
11	23	F	0.5ppd	x	x	S2b
12	43	F	20 pack-years	x	x	S2b
13	50	M	52.5 pack-years	x	x	S2b
14	57	M	15 pack-years	x	x	S2b

Figure S1. *S1P2* and *ACTA-2* mRNA Expression in HLMVEC. **(a)** Relative *S1P2* mRNA expression HLMVEC isolated from non-smokers (n=6) and smokers (n=5), measured by RTqPCR, normalized to the housekeeping gene H18S using the $2^{-\Delta\Delta CT}$ method and then expressed as a fold-change versus mean control (non-smoker) expression. $p=0.72$; 2-tailed unpaired Student t-test. **(b)** Relative *ACTA-2* mRNA expression in HLMVEC isolated from non-smoker (n=6) and smoker (n=5) donors, measured as in (a). $p=0.12$; 2-tailed unpaired Student t-test. Each data point represents an individual donor; horizontal lines are mean ± SEM.

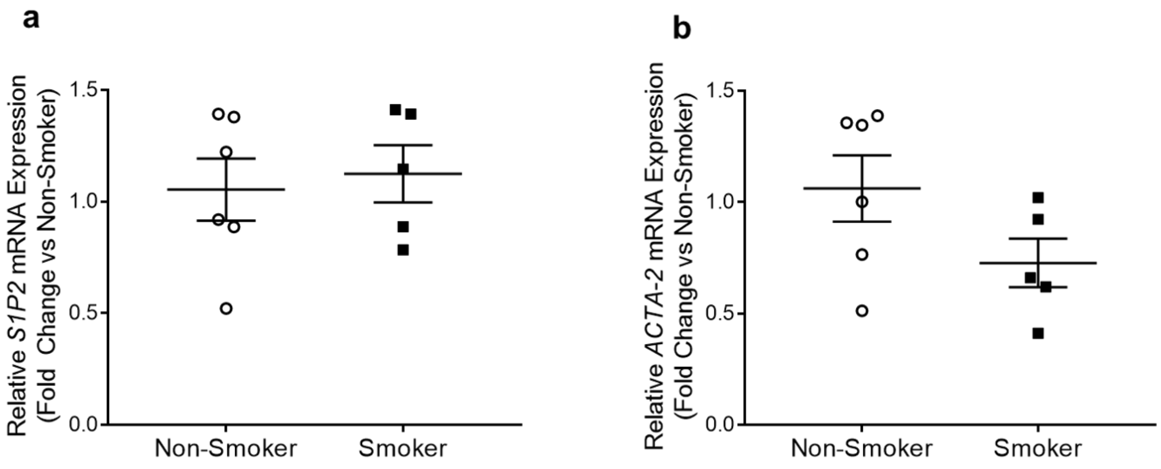

Figure S2. S1P1 in Human Lung Parenchyma. **(a)** Representative images focusing on alveolar areas using immunofluorescence performed on human non-smoker (NS) and smoker (Sm) fixed lungs, using antibodies against S1P1 (red), CD31 (green), and DAPI nuclear staining (blue). Inset (white box) shown at higher magnification for Sm lung. Images were captured at 200X; scale bar is 100 µm; scale bar in the magnified inset-image is 50 µm. White arrows are endothelial cells expressing S1P1, identified as positive for S1P1, CD31, and DAPI within a single cell. **(b)** S1P1 abundance in human alveolar tissue was quantified as the proportion (%) of CD31-positive cells positive for S1P1 in 11 non-smokers and smokers. 1 slide was prepared per individual and 5 alveolar areas were analyzed per slide. Each data point represents an individual donor. p=0.75; 2-tailed unpaired Student t-test. Horizontal lines are mean ± SEM.

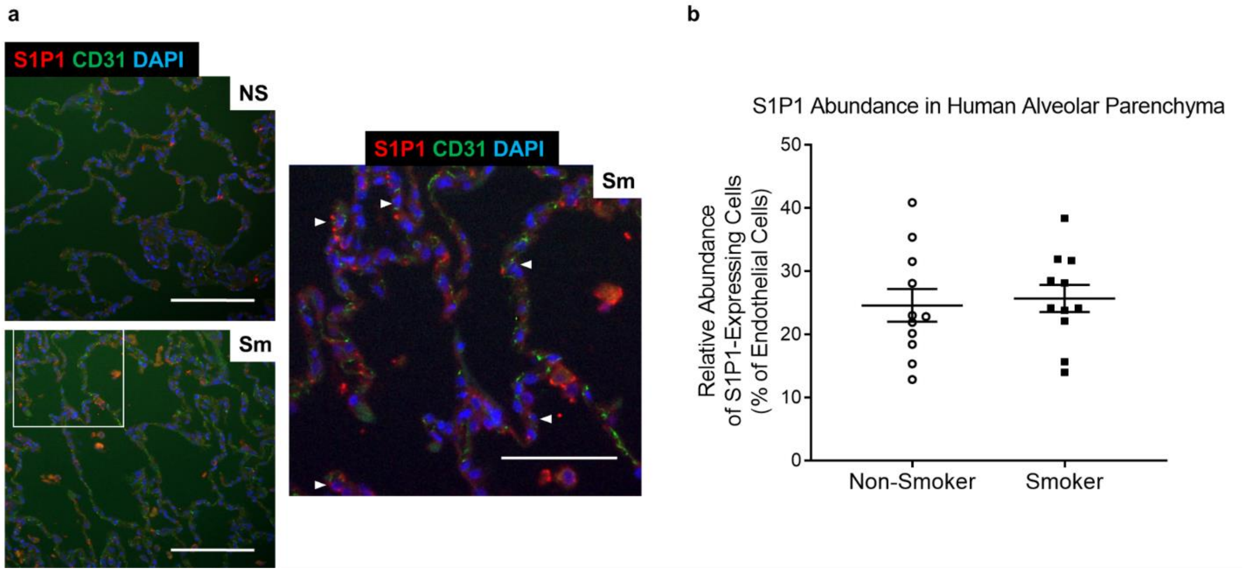

Supplement: Supplementary file 1 [file cells-10-01200-s001.zip › cells-1197867-SI.pdf]
